# Systemic disorders and the prognosis of stroke in Congolese patients: a cross-sectional study

**DOI:** 10.4314/gmj.v54i4.4

**Published:** 2020-12

**Authors:** Marc Tshilanda, Ulrick S Kanmounye, Remy Kapongo, Michel Tshiasuma

**Affiliations:** 1 Department of Internal Medicine, Centre Hospitalier Mère-Enfant (CHME) Monkole, Kinshasa DR Congo; 2 Faculty of Medicine, Université Notre-Dame du Kasayi, Kananga, Democratic Republic of Congo; 3 Faculty of Medicine, Université de Kinshasa, Kinshasa, Democratic Republic of Congo; 4 Research Department, Association of Future African Neurosurgeons, Yaounde, Cameroon

**Keywords:** Comorbidity, Democratic Republic of Congo, prognosis, sex differences, stroke

## Abstract

**Objectives:**

Stroke is one of the leading causes of death, disability, and dementia in developing countries. Our study aimed to evaluate the systemic disorders associated with mortality in patients admitted within 72 hours of the initial stroke event.

**Setting:**

The study took place at a tertiary hospital in Kinshasa.

**Participants:**

Patients admitted within 72 hours of the initial stroke event.

**Interventions:**

This cross-sectional study consisted of a retrospective review of stroke patient records from January 2016 to December 2018. The Pearson-Chi square test and odds ratios were calculated with a threshold of significance of 0.05.

**Main outcome measures:**

Mortality

**Results:**

We recruited 114 cases. The mean age was 61.8 ± 2.4 years, and the sex ratio was 1.78 in favor of men. Hypertension (76.3%), dyslipidemia (71.1%), and diabetes mellitus (58.8%) were the most frequent comorbidities. Most patients had hypoxia (85.9%), hypertension (82.4%), hyperglycemia (57.8%), and fever (28.1%). We registered thirty-two deaths (28.1%): 20 (62.5%) from the ischemic strokes, and 12 (37.5%) from hemorrhagic strokes. Systemic disorders with the worst prognosis during were arterial hypotension (OR=3.87, p >0.001), and fever (OR =1.56, p = 0.047).

**Conclusion:**

Arterial hypotension and fever adversely affect stroke patient outcomes, and strokes are responsible for high mortality in Congo.

**Funding:**

Not applicable

## Introduction

In 2017, strokes killed 6.2 million individuals worldwide; 87% of these deaths were registered in low and middleincome countries.^1^ The number of stroke cases in Africa has risen over the past three decades.^2^–^4^ There are more than 500.000 new stroke cases and 2 million stroke survivors in Africa.^2^ The fastest increase has been in the Democratic Republic of Congo (DRC), where the percentage change in incidence over 20 years is 27.8%.^5^ Strokes adversely affect the health of both female and male, young and old adults in DRC. They are responsible for 1.45% of total DALYs in the 15–49 age group and 6.57% of total DALYs in the 50–69 age group.^6^

Stroke intrahospital mortality in the DRC is 57%, and in an attempt to halt the epidemiological progression of strokes, the Congolese government has developed a national plan to prevent and manage strokes promptly.^7^,^8^,^9^

Several factors influence stroke patients' prognosis and should be identified and considered when deciding on the appropriate management strategy. Systemic disorders complicate stroke patients' management because they favor cerebral lesions factors and the deterioration of cerebral function.^5^,^7^,^8^

This study aims to identify the systemic disorders associated with mortality among patients admitted within 72 hours of the initial stroke event.

## Methods

### Ethics

This retrospective cross-sectional study was conducted after the authors were granted ethical approval N^o^ CHME/CIE/112/2019 from the Institutional Review Board of Centre Hospitalier Monkole (CHME). Data were extracted from the electronic patient records of stroke admissions at the emergency department.

### Study setting

CHME is a 400-bed reference hospital in the capital of DRC, Kinshasa, with a catchment area of 500,000 people.^10^

### Definitions

Stroke was defined as “rapidly developing clinical signs of focal (at times global) disturbance of cerebral function, lasting more than 24 hours or leading to death with no apparent cause other than that of vascular origin” 11 confirmed with a head computed tomography (CT) or magnetic resonance imaging (MRI).

Hypertension was defined as a systolic blood pressure ≥140 mmHg or a diastolic ≥ 90 mm Hg for up to 72 h after the stroke event, a documented history of hypertension, or the use of antihypertensive drugs before the stroke or more than 72 h after stroke and we did not apply OXVASC adjustments for blood pressure rises.^12^ Diabetes was defined as fasting blood glucose level ≥7.0 mmol/L, documented history of diabetes, or drug use for diabetes. In addition, we defined dyslipidemia as a fasting total cholesterol ≥5.2 mmol/L, HDL cholesterol ≤1.03 mmol/L, LDL cholesterol ≥3.4 mmol/L, or triglyceride ≥1.7 mmol/L. A cardiologist evaluated all our patients for cardiac disease, and we defined obesity using the body-mass index (BMI). Finally, alcohol consumption and smoking were defined based on patient history.

Economic status was defined using the hospital's three-tier classification based on locally sourced data. The three-tier classification takes into account the family's monthly income and financial responsibilities. It classifies the family based on local historical data into low income (percentiles 1–33), middle income (percentiles 34–66), and high income (percentiles 67–99). This classification is then used to bill families and avoid catastrophic financial expenditures. The hospital's social workers always visit the patients' neighborhoods and families to ensure that their declarations are authentic.

### Data collection and analysis

All patients admitted between January 1, 2016, to December 31, 2018, with a clinical suspicion of stroke and a head CT scan or MRI confirming a stroke were included.

Sociodemographic, clinical, radiologic, and laboratory data were collected using a standardized data collection tool. The authors ran descriptive data analyses together with Pearson-Chi square test and odds ratio. Also, the confidence intervals and the P-values were calculated when appropriate. All the analyses were done using SPSS v25.0 (IBM, New York).

## Results

From January 2016 to February 2018, strokes constituted 4.2% of all emergency department admissions (n=2692). Of the 114 patient admissions, most were male (Sex ratio 1.78), middle-income earners (55.3%), and the average age of patients was 61.8 years (SD=2.4) (Table 1)

**Table 1:** Sociodemographic of stroke patients from 2016 to 2018

Characteristic	2016	2017	2018	Total	*P*-value
**Age (years)**	65.2 ± 5.6	60.8 ± 3.1	59.5 ± 1.6	61.8 ± 2.4	0.021
**Sex**					
**Male**	27 (72.9%	24 (55.8%)	22 (67.7%)	73 (64.0%)	
**Female**	10 (27.1%)	19 (44.1%)	12 (35.3%)	41 (36.0%)	
**Economic status**					0.782
**Low-income**	16 (43.2%)	12 (27.9%)	10 (29.4%)	38 (33.3%)	
**Middle-income**	15 (40.5%)	28 (65.1%)	20 (58.8%)	63 (55.3%)	
**High-income**	6 (16.3%)	3 (7.0%)	4 (11.8%)	13 (11.4%)	

More than half (64.1%) of the stroke admissions were referrals, and limb weakness was the dominant symptom in 69 patients (60.5%). Hypertension (76.3%), dyslipidemia (71.1%), and diabetes mellitus (58.8%) were the most frequent comorbidities. Our patients' mean BMI was 28.2 Kg/m^2^, the mean systolic blood pressure at admission was 166.6 mmHg, and the average Glasgow Coma Scale was 12.5.

Sixty-four patients (56.1%) presented with hemiplegia, and most (71.8%) were right-sided. Forty-two (36.8%) patients had hemiparesis, of which 54.8% were rightsided (Table 2). further describes the clinical features of stroke patients.

**Table 2:** Clinical characteristics of Congolese patients admitted within 72 hours of the initial stroke event

Characteristic	Frequency	P-value
Presenting symptom		0.387
Coma	26 (22.8%)	
Limb weakness	69 (60.5%)	
Aphasia	10 (8.8%)	
Agitation and behavioral disorders	5 (4.4%)	
Associated diseases		-
Hypertension	87 (76.3%)	
Diabetes mellitus	67 (58.8%)	
Heart rhythm disorders	12 (10.5%)	
Valvulopathy	8 (7.0%)	
Ischemic heart disease	13 (11.4%)	
Dyslipidemia	81 (71.1%)	
Chronic alcoholism	32 (28.1%)	
Active smoking	7 (6.1%)	
Previous stroke episode	11 (9.6%)	
Pupils		0.033
Normal	87 (76.3%)	-
Anisocoric	14 (12.3%)	-
Bilateral myosis	5 (4.4%)	-
Bilateral mydriasis	8 (7.0%)	-
Hemiplegia	N=64	0.0473
Left	18 (28.2%)	-
Right	46 (71.8%)	-
Hemiparesis	N=42	0.0217
Left	19 (45.2%)	-
Right	23 (54.8%)	-
No motor deficit	8 (7.0%)	0.052

### Systemic disorders

Most (85.9%, n=98) patients had hypoxia, systolic hypertension (82.4%, n=94), and high blood glucose levels (57.8%, n=66). Fewer (28.1%, n=32) patients had a fever, and only 16 (14.0%) had no systemic disorders. The odds of death were higher among patients with hypotension (OR=3.52), fever (OR=1.50), hypoxia (OR=0.11), and systolic hypertension (OR=0.01) (Table 3).

**Table 3:** Regression analysis of the factors affecting in-hospital stroke patient mortality

Factor	Frequency	OR (95% CI)	P-value	OR (95% CI)	P-value
Hyperglycemia	66 (57.8%)	0,14 (0,03–0.06)	0.03	-	-
Hypoglycemia	14 (12.2%)	1.03 (0.3–3.56)	0.05	-	-
Hypertension	94 (82.4%)	0,01 (0.0–0.05)	0.000	-	-
Hypotension	12 (10.5%)	3.52 (1.12–5.92)	0.001	3.87 (2.07–8.67)	0.000
Fever	32 (28.1%)	1,50 (1.05–3.46)	0.002	1.56 (1.01–3.92)	0.047
Hypernatremia	4 (3.5%)	0,85 (0.09–1.61)	0.03	-	
Hyponatremia	16 (14.0%)	0,61 (0.12–1.09)	0.05	-	
Hypoxia	98 (85.9%)	0,11 (0.03–0.41)	0.000	-	
Cerebral edema	11 (9.6%)	2,63 (0.76–4.50)	0.007	3.12(1.96–10.57)	0.013
Other factors	4 (3.5%)	3,09 (1.21–4.88)	0.73	-	-

### Neuroimaging

Ischemic strokes were the most common stroke type (58.8%, n=67), and they were localized within the territories of the middle cerebral artery (47.8%), anterior cerebral artery (11.9%), and posterior cerebral artery (7.5%). On the other hand, hemorrhagic strokes (n=47) were located in the cerebral hemispheres (80.8%), cerebellum (17%), and brainstem (4.3%). Nineteen (40.4%) hemorrhagic stroke patients had an intraventricular hemorrhage while 14 (29.7%) had a subarachnoid hemorrhage. Eleven patients (9.6%) had a midline shift from brain edema (Table 4). Radiological cerebral edema multiplied the odds of having an unfavorable outcome by 2.83 (Table 3).

**Table 4:** Neuroimaging findings of stroke patients in the Democratic Republic of Congo

Finding	Frequency	*P*-value
Ischemic stroke	N=67(58.8%)	0.0412
Anterior cerebral artery territory	8 (11.9%)	
Middle cerebral artery territory	32 (47.8%)	
Posterior cerebral artery territory	5 (7.5%)	
Undetermined	12 (17.9%)	
Vertebrobasilar artery territory	7 (10.4%)	
Hemorrhagic stroke	N=47 (41,2%)	0.0173
Cerebral hemispheres	38 (80.8%)	
Interhemispheric commissures	22 (46.8%)	
Cerebellum	8 (17.0%)	
Brainstem	2 (4.3%)	
Intraventricular hemorrhage		
Subarachnoid hemorrhage	19 (40.4%)	
14 (29.7%)		

### Treatment and Outcome

Fifty-nine (88.1%) ischemic stroke patients were given low molecular weight heparin or another anticoagulant (11.9%) to prevent early neurologic deterioration. All the patients with intraventricular hemorrhage were treated surgically (external ventricular drain). Most (74.5%) patients treated medically had a favorable evolution. Twenty (29.9%) ischemic stroke patients died, while twelve (25.5%) hemorrhagic stroke patients died.

## Discussion

### Referrals

Although strokes were a small percentage of admission at the emergency department, they had a high mortality rate. Other African studies have reported similar stroke hospital prevalence and mortality rates. 9,13 These findings confirm that the burden of stroke is disproportionate in low- and middle-income countries, especially in deaths. Given our current understanding of ischemic and hemorrhagic strokes, these mortality rates are alarming. As the saying goes, “brain is time.” Yet, a significant proportion of patients were referrals. This is concerning because the DRC lacks a formal stroke referral system or an adequate pre-hospital network. Future studies should evaluate the stroke patient referral patterns and delays in reaching and receiving timely care.

Furthermore, we must quantify excess stroke-related deaths due to a delay in reaching and receiving care. This information can be used to design health systems strengthening efforts that will avert stroke-related deaths in low-resource settings.

### Age and Sex differences

The average age of patients in our study was similar to those of other African stroke studies. These studies found mean ages between 59–64 years.^14^–^17^ The life expectancy in the DRC is 60.03 years 7,8 only a couple of years away from the average age of Congolese stroke patients.

While this might be a coincidence, we posit that stroke contributes more to mortality among Congolese aged 50–69 than previously thought.

In our study, 64% of stroke patients were males. The male sex has been linked with a higher predisposition to strokes 14,17–20, but other studies have found that the female sex is more affected.^15^,^21^,^22^ These differences might be due to cumulative risk differences. While men have a higher risk than females of the same age, women tend to live longer and are exposed for more extended periods.^23^ The Framingham female stroke patients were older than men when they suffered their first stroke event.^24^ Therefore, stroke populations with a higher average age for index stroke events tend to have a female predominance. In comparison, studies with a lower average age tend to have a male predominance.

Our study noticed a decrease in the mean age over the three years from 65.2 in 2016 to 59.5 in 2018. This might explain the male predominance in our study. It is important to consider other factors, however. Some studies have identified sex differences in the etiology, presentation, treatment, and outcome of strokes.^23^,^25^ Left ventricular hypertrophy, for example, is more prevalent among female African and hemorrhagic stroke patients. At the same time, atrial fibrillation is more common among ischemic stroke patients.^26^ Also, the higher prevalence of hemorrhagic and lacunar strokes among Africans has been attributed to a higher prevalence of risk factors such as hypertension and left ventricular hypertrophy.^5^ A higher prevalence of hemorrhagic strokes coupled with more severe and a higher prevalence of comorbidities might mean that African female patients are more likely to die from a stroke and, as a result, less likely to be admitted to the emergency department. These theories require further investigation.

### Systemic Disorders

More than half of our patient population either had hypertension, dyslipidemia, or diabetes. These diseases are known stroke risk factors, which can avert stroke events when identified and managed.^27^

Given the role of comorbidities, it is essential to invest in primary health care to diagnose and manage stroke risk factors before the first stroke event. We found that hypotension (OR=3.52) and fever (OR=1.50) had the most significant association with mortality. They have both been identified as factors of poor prognosis in stroke patients.^28^ They increase the odds of stroke-related death by facilitating a cascade of tissue ischemia, excitotoxicity, mitochondrial dysfunction, and cell death.^29^

Of all the factors favoring brain injury, hypoxia and hypotension are known to increase the morbidity and mortality of brain-injured patients considerably.^30^,^31^ To monitor changes and prevent these risk factors' adverse effects, brain-injured patients should ideally be managed by multidisciplinary neuro-intensive care (stroke) units equipped with invasive neuromonitoring devices.^32^ Unfortunately, this is not always possible in low-resource settings where there is a shortage of specialist workforce and a lack of invasive neuromonitoring devices. There are cheaper but less reliable modalities that can be used to monitor blood pressure, blood glucose, and oxygenation - blood pressure monitors/sphygmomanometers, blood glucose meters, and pulse oximeters. These modalities are labor-intensive (require regular measurements); however, they can give the medical team valuable information that will inform the management of patients admitted within 72 hours of the initial stroke event.

Twenty-six cases presented in a coma (22.8%), 10 (8.8%) had a speech disorder. Ndigue et al. 33 had 25% of patients presenting with a consciousness disorder, and 20.9% presented with speech disorder. The difference in the proportion of speech disorders could be attributed to the emergency department's delayed presentation. The most common stroke subtype was a middle cerebral artery ischemic stroke. Ischemic strokes secondarily cause lesions to adjacent penumbra regions, and in the middle cerebral artery, a notable region is that of speech. We can hypothesize that the delay in reaching the hospital was detrimental to Ndigue et al.'s 33 patients' functional outcome.

### Implications

The mortality rates in our cohort were above 20% in both ischemic and hemorrhagic stroke patients. All of our patients received contextually appropriate treatment. Their management was still made difficult by the lack of thrombolytic drugs, endovascular treatment, and open vascular surgery. In DRC, there are no catheterization labs; there are 34 intensivists and a mere eight neurosurgeons for 84 million inhabitants.^34^–^36^ The few hospitals in the country with functioning stroke units are overwhelmed by cases, and their resources limit their capacity.

This study highlights the need for investment in the stroke patient continuum of care from prevention to rehabilitation. The investments must concern all the aspects of the health system: workforce, infrastructure, funding, information management, service delivery, and governance. Only a holistic approach can lead to an improvement in patient outcomes.

### Limitations

We recognize a few limitations to our study. In this study, we used the WHO's definition of stroke – “rapidly developing clinical signs of focal (at times global) disturbance of cerebral function, lasting more than 24 hours or leading to death with no apparent cause other than that of vascular origin.” – and then confirmed the cases with a CT/MRI. As a result, we excluded patients with clinical signs of stroke and negative neuroimaging. This could constitute a significant bias. Next, we did not take into account the OXVASC adjustment when defining hypertension.^12^

## Conclusion

This investigation aimed to determine the factors associated with mortality of patients admitted within 72 hours of the index stroke event at CHME. Adult Congolese men are most affected by stroke, and ischemic strokes are more common than hemorrhagic strokes. Hypotension and fever significantly increase the odds of a patient dying and should be recognized and managed accordingly. There is a need to develop specialized stroke units and reorganize the current referral system in the DRC.
